# Many-body interactions in the Al–Cu system

**DOI:** 10.1016/j.dib.2017.10.002

**Published:** 2017-10-06

**Authors:** O.I. Gorbatov, Yu. N. Gornostyrev, P.A. Korzhavyi

**Affiliations:** a Department of Materials Science and Engineering, KTH Royal Institute of Technology, SE-100 44 Stockholm, Sweden; b Institute of Quantum Materials Science, Ekaterinburg 620107, Russia; c Nosov Magnitogorsk State Technical University, Magnitogorsk 455000, Russia; d Institute of Metal Physics, Ural Division RAS, Ekaterinburg 620219, Russia

## Abstract

The article contains computational data of many-body interactions in Al–Cu alloys, obtained using PAW-VASP calculations. Pairwise, three-site, and four-site interactions are presented. Mentioned data are relevant to the research article “Many-body mechanism of Guinier-Preston zones stabilization in Al–Cu alloys” (Gorbatov et al., 2017) [1].

**Specifications Table**

**Table:** 

Subject area	*Physics*
More specific subject area	*Condensed matter physics, Materials science*
Type of data	*Tables, figures*
How data was acquired	*Density functional theory calculations using the PAW-VASP method*
Data format	*Raw, calculated, analyzed*
Experimental factors	*N/A*
Experimental features	DFT calculations
Data source location	*Institute of Quantum Materials Science, Ekaterinburg 620107, Russia*
Data accessibility	Data are presented in this article

**Value of the data**

• This data allows other researchers to calculate and predict the phase stability in Al–Cu based alloys.
• The data can be used as input for further theoretical evaluation of properties and for the verification of the validity of other models and approaches.
• The data can be compared with other theoretical and experimental results.
• Many-body interactions in the same data format can be calculated for other alloys.

## 1 Data

The results of calculations are shown in Table 1, Table 2, Table 3. Tables contain atomic coordinates of clusters used for calculations. Effective interactions in dilute Al-Cu alloys are presented in Table 1 for pair interactions $V_{p}^{\left(2\right)}$ as a function of coordination shell number, in Table 2 for triplet $V_{t}^{\left(3\right)}$, and in Table 3 for quadruplet $V_{q}^{\left(4\right)}$ interactions for different atomic clusters. The calculated formation energies of n-site Cu clusters of type *ν* are presented in these tables as well. Moreover, coordinates of atoms in clusters are shown. The effective cluster interactions and formation energies of corresponding Cu clusters are presented for unrelaxed and relaxed atomic configurations. The Tables show that unrelaxed interactions converge very rapidly with respect to both the diameter of the cluster and the number of sites comprising it. Relaxed ECIs (containing the strain-induced contributions) remain to be short-ranged, but become essentially many-body to lower the energy of planar Cu clusters relative to that of “bulky” ones. At least four-body compact clusters have to be included in the cluster expansion to qualitatively reproduce the stability and morphology of GP zones in Al–Cu alloys.

**Table 1:** Effective pair interactions in dilute Al–Cu alloys $V_{p}^{\left(2\right)}$ (eV) as a function of coordination shell number p. Second column shows an index notation of pair clusters by the vector connecting the two atoms (in a/2 units). Coordinates of atoms in the pair clusters are given in units of lattice parameter a.

Pair, *p*	Index notation	Atom coordinates	Pair interactions $V_{p}^{\left(2\right)}$	
			Relaxed	Unrelaxed
1	〈110〉	(0.0, 0.0, 0.0); (0.5, 0.5, 0.0)	− 0.040	0.025
2	〈200〉	(0.0, 0.0, 0.0); (0.1, 0.0, 0.0)	− 0.005	0.008
3	〈211〉	(0.0, 0.0, 0.0); (0.5, 0.5, 1.0)	0.009	0.002
4	〈220〉	(0.0, 0.0, 0.0); (0.0, 1.0, 1.0)	0.006	− 0.004
5	〈310〉	(0.0, 0.0, 0.0); (1.5, 0.5, 0.0)	− 0.006	− 0.005
6	〈222〉	(0.0, 0.0, 0.0); (1.0, 1.0, 1.0)	0.009	0.003
7	〈321〉	(0.0, 0.0, 0.0); (0.5, 1.0, 1.5)	0.002	− 0.002
8	〈400〉	(0.0, 0.0, 0.0); (2.0, 0.0, 0.0)	− 0.005	− 0.003

**Table 2:** Effective triplet interactions $V_{t}^{\left(3\right)}$ (eV) and formation energies of three-site Cu clusters $E_{t}^{\left(3\right)}$ (eV) for different atomic clusters. Three-site clusters are denoted by indices $t_{1}t_{2}t_{3}$, where $t_{i}$ specifies the coordination shell radii separating every pair of atoms in these clusters. Coordinates of atoms in clusters are shown.

Triplet, t	Atom coordinates	$V_{t}^{\left(3\right)}$		$E_{t}^{\left(3\right)}$	
		Relaxed	Unrelaxed	Relaxed	Unrelaxed
111	(0.0, 0.0, 0.0); (0.5, 0.5, 0.0); (0.5, 0.0, 0.5)	− 0.037	− 0.018	− 0.082	0.093
112	(0.0, 0.0, 0.0); (0.5, 0.5, 0.0); (1.0, 0.0, 0.0)	0.031	0.005	− 0.115	0.053
113	(0.0, 0.0, 0.0); (0.5, 0.5, 0.0); (1.0, 0.5, 0.5)	0.007	0.006	− 0.078	0.047
114	(0.0, 0.0, 0.0); (0.5, 0.5, 0.0); (1.0, 1.0, 0.0)	0.011	0.002	− 0.084	0.044
123	(0.0, 0.0, 0.0); (0.5, 0.5, 0.0); (0.0, 0.0, 1.0)	− 0.006	0.003	− 0.030	0.032
133	(0.0, 0.0, 0.0); (0.5, 0.5, 1.0); (0.5, 1.0, 0.5)	0.002	− 0.001	− 0.025	0.031
125	(0.0, 0.0, 0.0); (0.0, 0.0, 1.0); (0.0, 0.5, 1.5)	0.002	− 0.001	− 0.053	0.029
224	(0.0, 0.0, 0.0); (0.0, 1.0, 0.0); (1.0, 0.0, 0.0)	0.003	0.004	− 0.007	0.008
134	(0.0, 0.0, 0.0); (0.5, 0.5, 1.0); (0.0, 1.0, 1.0)	− 0.002	0.004	− 0.023	0.020
233	(0.0, 0.0, 0.0); (0.0, 0.0, 1.0); (0.5, 1.0, 0.5)	0.002	0.003	0.010	0.009
135	(0.0, 0.0, 0.0); (1.0, 0.5, 0.5); (1.5, 0.5, 0.0)	0.004	0.001	− 0.041	0.022
136	(0.0, 0.0, 0.0); (0.5, 0.5, 0.0); (1.0, 1.0, 1.0)	0.000	0.003	− 0.022	0.028
334	(0.0, 0.0, 0.0); (1.0, 1.0, 0.0); (0.5, 0.5, 1.0)	− 0.004	− 0.001	0.027	0.001
145	(0.0, 0.0, 0.0); (1.0, 1.0, 0.0); (1.5, 0.5, 0.0)	0.002	0.001	− 0.041	0.016
137	(0.0, 0.0, 0.0); (0.5, 0.5, 0.0); (1.5, 1.0, 0.5)	0.002	0.001	− 0.031	0.024
155	(0.0, 0.0, 0.0); (1.5, 0.5, 0.0); (1.5, 0.0, 0.5)	− 0.002	0.000	− 0.050	0.017
237	(0.0, 0.0, 0.0); (0.5, 1.0, 0.5); (1.5, 1.0, 0.5)	− 0.001	0.000	0.006	0.008
147	(0.0, 0.0, 0.0); (1.0, 1.0, 0.0); (1.5, 1.0, 0.5)	0.000	0.001	− 0.032	0.019
246	(0.0, 0.0, 0.0); (0.0, 0.0, 1.0); (1.0, 1.0, 1.0)	− 0.002	0.000	0.012	0.007
345	(0.0, 0.0, 0.0); (1.0, 0.0, 1.0); (1.5, 0.5, 0.0)	0.000	0.001	0.009	− 0.007
444	(0.0, 0.0, 0.0); (1.0, 1.0, 0.0); (0.0, 1.0, 1.0)	− 0.003	− 0.001	0.022	− 0.010
255	(0.0, 0.0, 0.0); (0.0, 1.0, 0.0); (1.5, 0.5, 0.0)	0.001	0.002	− 0.018	− 0.003
157	(0.0, 0.0, 0.0); (1.5, 0.0, 0.5); (1.5, 0.5, 1.0)	− 0.002	0.000	− 0.041	0.019

**Table 3:** Effective quadruplet interactions $V_{q}^{\left(4\right)}$ (eV) and formation energies of four-site Cu clusters $E_{q}^{\left(4\right)}$ (eV) for different atomic clusters. Four-site clusters are denoted by indices $q_{1}q_{2}q_{3}q_{4}q_{5}q_{6}$, where $q_{j}$ specifies the coordination shell radii separating every pair of atoms in these clusters. Coordinates of atoms in the clusters are given.

Quadruplet, q	Atom coordinates	$V_{q}^{\left(4\right)}$		$E_{q}^{\left(4\right)}$	
		Relaxed	Unrelaxed	Relaxed	Unrelaxed
111111	(0.0, 0.0, 0.0); (0.5, 0.5, 0.0); (0.5, 0.0, 0.5); (0.0, 0.5, 0.5)	0.132	0.011	0.042	0.232
111112	(0.0, 0.0, 0.0); (0.5, 0.0, 0.5); (0.0, 0.5, 0.5); (0.0, 0.0, 1.0)	0.038	0.003	− 0.153	0.163
111122	(0.0, 0.5, 0.5); (0.5, 0.0, 0.5); (0.5, 1.0, 0.5); (1.0, 0.5, 0.5)	− 0.037	0.000	− 0.329	0.097
111113	(0.0, 0.0, 0.0); (0.5, 0.0, 0.5); (0.0, 0.5, 0.5); (0.5, 0.5, 1.0)	− 0.002	0.005	− 0.132	0.157
111123	(0.0, 0.0, 0.0); (0.5, 0.5, 0.0); (0.5, 0.0, 0.5); (0.0, 0.0, 1.0)	0.002	0.003	− 0.148	0.118
111133	(0.0, 0.0, 0.0); (0.0, 0.5, 0.5); (0.5, 0.5, 1.0); (0.5, 1.0, 0.5)	0.000	− 0.001	− 0.121	0.111
111134	(0.0, 0.0, 0.0); (0.0, 0.5, 0.5); (0.5, 0.5, 1.0); (0.0, 1.0, 1.0)	0.001	0.004	− 0.123	0.109
111224	(0.0, 0.0, 0.0); (0.5, 0.5, 0.0); (0.0, 1.0, 0.0); (1.0, 0.0, 0.0)	0.005	0.003	− 0.195	0.075
111233	(0.0, 0.0, 0.0); (0.5, 0.5, 0.0); (1.0, 0.5, 0.5); (0.5, 0.5, 1.0)	0.000	− 0.005	− 0.137	0.072
111225	(0.0, 0.0, 0.0); (0.5, 0.5, 0.0); (0.0, 1.0, 0.0); (0.5, 1.5, 0.0)	0.004	− 0.001	− 0.197	0.078
111334	(0.0, 0.0, 0.0); (0.5, 1.0, 0.5); (1.0, 0.5, 0.5); (1.0, 1.0, 0.0)	− 0.002	− 0.005	− 0.060	0.083
112234	(0.0, 0.0, 0.0); (1.0, 0.0, 0.0); (1.0, 0.5, 0.5); (1.0, 1.0, 0.0)	0.000	0.002	− 0.101	0.051
111245	(0.0, 0.0, 0.0); (0.5, 0.5, 0.0); (1.0, 1.0, 0.0); (1.5, 0.5, 0.0)	0.004	0.003	− 0.166	0.071
112334	(0.0, 0.0, 0.0); (0.5, 0.5, 0.0); (1.0, 1.0, 0.0); (0.5, 0.5, 1.0)	− 0.002	0.001	− 0.059	0.052
111345	(0.0, 0.0, 0.0); (1.5, 0.5, 0.0); (1.0, 1.0, 0.0); (1.0, 0.5, 0.5)	0.001	0.000	− 0.077	0.081
122334	(0.0, 0.0, 0.0); (0.0, 0.5, 0.5); (1.0, 0.0, 0.0); (1.0, 1.0, 0.0)	0.000	0.002	− 0.025	0.030
113334	(0.0, 0.0, 0.0); (0.5, 0.5, 1.0); (0.5, 1.0, 0.5); (1.0, 1.0, 0.0)	0.000	− 0.004	− 0.051	0.042
222244	(0.0, 0.0, 0.0); (0.0, 1.0, 0.0); (1.0, 0.0, 0.0); (1.0, 1.0, 0.0)	0.001	0.002	− 0.020	0.010
113444	(0.0, 0.0, 0.0); (0.5, 0.5, 0.0); (1.0, 1.0, 0.0); (0.0, 1.0, 1.0)	0.001	0.001	− 0.057	0.034
222444	(0.0, 0.0, 0.0); (1.0, 0.0, 0.0); (1.0, 1.0, 0.0); (1.0, 0.0, 1.0)	0.000	0.000	− 0.004	0.002
133444	(0.0, 0.0, 0.0); (0.5, 0.0, 0.5); (0.0, 1.0, 1.0); (1.0, 1.0, 0.0)	0.000	0.001	0.006	0.013

## 2 Methods

The energy of an Al–Cu alloy is represented in the form of a cluster expansion by the following Ising-type configurational Hamiltonian [2,3]:

(1) $$H=\frac{1}{2^{2}\cdot 2!}\sum_{i,j\in p}V_{p}^{\left(2\right)}\sigma_{i}\sigma_{j}+\frac{1}{2^{3}\cdot 3!}\sum_{i,j,k\in p}V_{t}^{\left(3\right)}\sigma_{i}\sigma_{j}\sigma_{k}+\frac{1}{2^{4}\cdot 4!}\sum_{i,j,k,l\in q}V_{q}^{\left(4\right)}\sigma_{i}\sigma_{j}\sigma_{k}\sigma_{l}$$

where $V_{p}^{\left(2\right)}$, $V_{t}^{\left(3\right)}$, and $V_{q}^{\left(4\right)}$ are two-, three- and four-body interactions for the corresponding two-, three-, and four-site clusters denoted as p, *t*, and *q*. Further, $\sigma_{j}$ is a spin variable that can take on values + 1 or − 1 depending on which type of atom (Al or Cu, respectively) occupies site *j* [2].

**Fig. 1:**
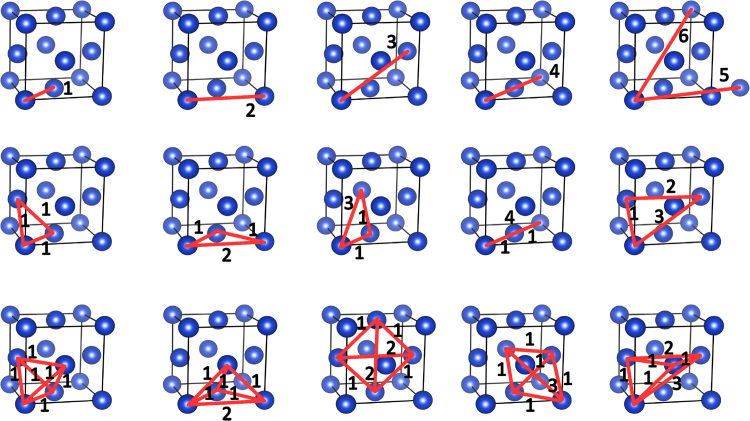
A schematic representation of atomic clusters. Two-site (1, 2, 3, 4, 5, 6), three-site (111, 112, 113, 114, 123), and four-site (111111, 111112, 111112, 111113, 111123) clusters are indicated by red lines connecting each pair of atoms in the cluster. Each line is indexed by the corresponding coordination shell number.

Two-site clusters are denoted by the coordination shell number *p* corresponding to the vector connecting the two atomic sites, or by an index notation 〈hkl〉 of the corresponding family of vectors, $\frac{a}{2}\langle \mathrm{hkl}\rangle$, where *a* is the lattice parameter; the index notation is commonly used in experimental studies of atomic short-range order. Geometry of multisite clusters can be described by enumerating the vectors separating every two atoms in these clusters [3]. Therefore, three-site and four-site clusters are denoted here, respectively, by indices $t_{1}t_{2}t_{3}$ and $q_{1}q_{2}q_{3}q_{4}q_{5}q_{6}$, where $t_{i}$ and $q_{j}$ specify the coordination shell radii separating every pair of atoms in these clusters. Some examples of two-site, three-site, and four-site clusters are shown in Fig. 1. It should be noted that in this work, for simplicity, the indices have been sorted in the ascending order.

The effective cluster interactions (ECI) are determined in this work from a set of total energy calculations for supercells, each representing a single configuration (case of site occupancy by Al and Cu atoms) of a cluster ν in {p,t,q,…} in the otherwise pure Al matrix. Then an n-body interaction for an n-site cluster ν is calculated as

(2) $$V_{\nu }^{\left(n\right)}=\sum_{{\left\{\sigma \right\}}_{\nu }}E({\left\{\sigma \right\}}_{\nu })\prod_{i\in \nu }\sigma_{i}$$

where $E\left({\left\{\sigma \right\}}_{\nu }\right)$ is the total energy of a supercell containing cluster ν in the configuration ${\left\{\sigma \right\}}_{\nu }$. The product of *n* spin variables in Eq. (2) equals 1(−1) whenever an even (odd) number of Cu atoms is involved in the cluster ν. Eq. (2) allows one to calculate the different ECI directly and independently from each other using a large enough supercell, without the need to do a least-square fitting procedure to calculate the ECI or cross-validation analysis to judge which interactions are important and which are negligible.

**Fig. 2:**
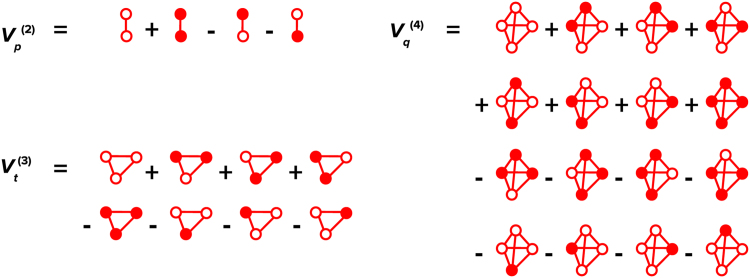
Scheme for calculations two- ($V_{p}^{\left(2\right)}$), three- ($V_{t}^{\left(3\right)}$), four-body ($V_{t}^{\left(4\right)}$) interactions. An *n*-body interaction is calculated as the difference between energies of supercells each containing an even number of Cu atoms and energies of supercells each containing an odd number of Cu atoms in the n-site cluster. Filled circles correspond to Cu atoms in the Al matrix.

The procedure of calculating effective cluster interactions is exemplified in Fig. 2 showing all cases of site occupancy (configurations ${\left\{\sigma \right\}}_{\nu }$ of two-, three-, four-body clusters ν={p,t,q} by two atomic species. The energies of these configurations enter the sum in Eq. (2) with the plus (minus) sign in $\frac{1}{2}$ of the cases. As a consequence, the ECI calculated using Eq. (2) for a many-body cluster contains just the *excess* energy in addition to that contained in the lower-order ECIs representing the constituent parts of the cluster.

The formation energy of an n-site solute (Cu) cluster of ν-type is defined as

(3) $$E_{\nu }^{\left(2\right)}=E_{\nu }+\left(n-1\right)E_{0}-nE_{1}$$

where the E terms are the calculated total energies of supercells. Subscript 0 denotes a supercell without defects, subscript 1 denotes a supercell containing a single solute (Cu) atom, while subscript ν denotes a supercell containing an *n*-site solute cluster ν. It should be noted that for two-site clusters, n=2, one has $E_{\nu }^{\left(2\right)}=V_{\nu }^{\left(2\right)}$.

The effective interactions were calculated using a 256-site supercell obtained by 4×4×4 repetition of the 4-atom fcc unit cell. To keep the cubic symmetry of the underlying fcc lattice, which is preserved on average in real alloys, the supercell translation vectors were kept fixed. The calculations were performed at the fcc lattice constant of *a* = 4.05 Å. The total energy calculations for the supercells were performed using the projector augmented wave (PAW) method [4] as implemented in VASP package [5]. The generalized gradient approximation and a plane wave basis with a cutoff energy of 350 eV were used in the PAW calculations. The integration over the Brillouin zone was done using the 4×4×4 Monkhorst-Pack grid. The convergence criterion for total energy was 10^− 5^ eV/cell; the forces were relaxed below 10^− 2^ eV/Å during internal structural optimization. More computational detail can be found in Ref. [1].
